# Food Acquisition Locations and Food Groups Acquired According to Levels of Food Insecurity in Brazil

**DOI:** 10.3390/ijerph21121577

**Published:** 2024-11-27

**Authors:** Roberta Teixeira de Oliveira, Paulo César Pereira de Castro Junior, Yoko Ametista Carvalho Suéte Matos, Aline Alves Ferreira, Rosana Salles-Costa

**Affiliations:** 1 Postgraduate Program in Nutrition, Josué de Castro Nutrition Institute, Federal University of Rio de Janeiro (UFRJ), Rio de Janeiro 21941-590, Brazil; 2 Department of Social and Applied Nutrition, Josué de Castro Nutrition Institute, Federal University of Rio de Janeiro (UFRJ), Rio de Janeiro 21941-590, Brazil

**Keywords:** food security, food insecurity, socioeconomic factors, food environments, Brazil

## Abstract

Food insecurity (FI) is a persistent issue in Brazil, with significant disparities existing across the country’s macroregions. This study investigated the food acquisition locations and types of foods purchased at different levels of FI, utilizing data from 57,920 households in the 2017–2018 Brazilian Household Budget Survey. Food acquisition locations were grouped into supermarkets, small markets, street fairs/fruit and vegetable stores/public markets, and others. Foods were categorized into 12 groups: rice, beans, vegetables, fruits, red meats, fish, poultry, eggs, milk and dairy products, bread, cookies, and sugary drinks. Supermarkets are the primary source of food in Brazil. However, in the North and Northeast regions, small markets are more frequently used across all levels of FI. Nationally, most food groups are predominantly purchased at supermarkets. Nevertheless, households experiencing moderate or severe FI rely more on small markets for essential items such as rice, beans, and proteins, as well as ultraprocessed foods. These findings highlight the need for public policies that improve food access for vulnerable populations and address regional inequalities. Enhancing access and ensuring food security across different regions is essential to promoting equitable and healthy diets throughout the country.

## 1. Introduction

Recently, the State of Food and Nutrition Security in the World (FNS) reported that the global prevalence of moderate or severe food insecurity (FI) remains far above pre-COVID levels (FAO, 2024) [1]. According to the FAO, the levels of FI have remained virtually unchanged. In 2023, an estimated 28.9% of the global population had moderate or severe levels of FI, meaning that they did not have regular access to adequate food.

In Brazil, access to food of sufficient quality and quantity is a human right guaranteed by the Organic Law of Food and Nutritional Security (LOSAN) [2]. Failure to realize this right indicates the presence of FI, a key indicator for assessing food and nutrition security (FNS) in population studies [3]. The concept of FNS adopted in Brazil [4] encompasses various dimensions. To ensure access to adequate and healthy food, both the availability of financial resources and physical access to food must be considered. This can be evaluated based on proximity to places where food is available for purchase [5,6].

Access to food is influenced by the characteristics of the food environment, which encompasses the physical, economic, political, and sociocultural contexts in which consumers interact with the food system to obtain, prepare, and consume food [7]. The physical aspects of the food environment, including the type and location of food outlets, as well as the ease of access to food products, make up what is known as the community or retail food environment [8,9]. Recent studies indicate that food environments are closely related to FNS and FI [10,11], as the way food is marketed is just as important as regular access to food of adequate quantity and quality.

Since 2013, Brazil has experienced a considerable increase in the level of FI, especially in severe FI, which worsened throughout 2018 and presented a rising trend in the following years [12]. In contrast, food security has shown a declining pattern among families, influenced by the political crisis and the dismantling of public policies promoting FNS, particularly since 2016 [12,13]. These factors have compromised the population’s access to adequate and healthy food.

Despite the importance of analyzing the relationship between FI and food environments, few studies have investigated this relationship in connection with food acquisition locations in Brazil. This topic is highly important because the access to and availability of food are directly linked to the country’s socioeconomic and geographical characteristics, which can influence dietary patterns and either exacerbate or alleviate FI. Therefore, this study investigated the places of food acquisition, and the types of food acquired according to different levels of FI in Brazil, with the aim of contributing to a better understanding of these dynamics within a national context.

## 2. Materials and Methods

### 2.1. Ethical Statement

According to Resolution No. 510 of the National Research Ethics Commission (CONEP), dated 7 April 2016, research via publicly available secondary data that do not identify research participants does not require CONEP approval. The information contained in the POF microdata does not allow for the identification of the studied households, as specific data about each household, such as addresses, phone numbers, and census sector numbers, are omitted. Thus, approval was not required for this study (available at https://bvsms.saude.gov.br/bvs/saudelegis/cns/2016/res0510_07_04_2016.html, accessed on 5 March 2021).

### 2.2. Data Source, Population, and Sampling

In this cross-sectional, population-based study, microdata from the 2017/2018 Household Budget Survey (POF), which were provided by the Brazilian Institute of Geography and Statistics (IBGE), were evaluated. The POF is a nationally representative household survey that provides information on household budget composition and offers data and studies on the nutritional profile of the population [14]. The POF is the only survey that assesses both food acquisition dimensions and FI among Brazilian families, and the 2017/2018 edition is the most recent dataset available in which these aspects are evaluated concurrently.

The survey followed a two-stage cluster sampling design with geographical stratification (Brazilian macroregions and the Federal District, urban/rural situation). Information was collected directly from 55,920 permanent private households, considering each family a consumption unit (a single resident or group of residents), selected between July 2017 and July 2018. This information was used to account for seasonal variations in income, prices, and food purchases. The data were collected by trained interviewers who met with the residents in person and gathered the information over consecutive days during a nine-day period, using portable computers for data recording and entry [14]. The interviews were evenly distributed across the four quarters of the survey to capture variations in expenses throughout the year, such as seasonal changes in income and food acquisitions within the strata.

Data were collected via questionnaires organized into blocks based on the type of information: POF 1 (Household and Resident Characteristics Questionnaire); POF 2 (Collective Acquisition Questionnaire); POF 3 (Collective Acquisition Diary); POF 4 (Individual Acquisition Questionnaire); POF 5 (Individual Work and Income Questionnaire); POF 6 (Living Conditions Assessment); and POF 7 (Personal Food Consumption Block).

This study evaluated records from the households’ collective food acquisition diaries (POF 3), where all information regarding food and beverage purchases was recorded for seven consecutive days by all household members, either by the residents themselves or by the IBGE interviewer if necessary. In addition to the items purchased for the families’ consumption (product quantity; unit of measure, such as weight or volume; type of product; and value in BRL), the places of acquisition (supermarket, bakery, snack bar, butcher, fish market, street market, pharmacy, etc.) were recorded in the diaries.

### 2.3. Places of Food Acquisition

The different places of food acquisition reported in the 2017–2018 POF were organized into four groups on the basis of the nature of the main products available and specific marketing characteristics. For example, ‘supermarket’ and ‘hypermarket’ can be defined as self-service stores offering a wide variety of food, ranging from fresh produce to ultraprocessed products, where items are organized by department. ‘Small market’ can be defined as a small store where food products occupy most of the shelf space, in addition to household items, although the inventory is limited. ‘Street fairs/fruit and vegetable stores’ can be defined as stores that primarily sell fruits and vegetables [15]. The classification was based on and adapted from groupings proposed by Costa et al. [16], using data from the 2002–2003 POF, and Machado et al. [17], using data from the 2008–2009 POF, corresponding to the following groups: Group 1 (supermarkets [supermarkets, hypermarkets, and wholesalers]); Group 2 (small markets [markets, mini-markets, grocery stores, bakeries, warehouses, emporiums, and candy and natural product stores]); Group 3 (street fairs/fruit and vegetable stores/public markets); and Group 4 (other places [gas station convenience stores, pharmacies, schools, churches, food trucks, snack bars, restaurants, pastry shops, coffee shops, and delivery services]). Group 4 was not included in the data analyses of this study, as the percentage of food acquired from these locations was less than 10%.

### 2.4. Food Groups

The foods recorded in a household’s collective acquisition diary were grouped into rice, beans, vegetables and greens, fruits, red meats, fish, poultry, eggs, milk and dairy products, breads, biscuits, and sugary beverages. This classification was based on adaptations of the food subgroups previously established by the POF in its most recent edition (IBGE, 2020) [18]. Table 1 presents descriptions of the analyzed food groups.

### 2.5. Household Food Insecurity

The Brazilian Food Insecurity Scale (EBIA) was used in the POF to classify households into food security or levels of FI (mild, moderate, or severe). The EBIA consists of a total of 14 questions addressing family access to food in terms of quantity and quality, with increasing severity, ranging from concerns about running out of food before being able to buy or receive more, to cases where residents had only one meal a day or went without eating for an entire day because there had been no money to buy food in the past three months. Of these questions, 8 pertain to residents aged 18 or older, while the remaining 6 questions are for residents under 18 [19]. When all the questions are answered negatively by the household head, the family is classified as food-secure. In contrast, families are classified into three levels of FI severity (mild, moderate, or severe) according to the sum of positive responses [19,20]. In this study, families were categorized into three groups: food secure, mildly food insecure, and moderately/severely food insecure. The moderate and severe levels were combined to more accurately compare the severity of lack of access to food among families, following the approach of other studies [21,22].

### 2.6. Covariates

The choice of sociodemographic indicators related to FI estimated by EBIA was based on the systematic review published by Lignani et al. [3]. According to the authors, family income assumed a central role in the mediation between several sociodemographic indicators and FI. Additionally, the area of residence (urban/rural) presents a dual consideration, since both areas can have a direct relationship with food security or the severity of FI. Gubert et al. debated the relationship of the protective factor related to living in rural areas against FI due to the greater possibility of food production for family consumption and lower expenses for transportation, clothing, and others [23]. In another article, Gubert considered that families living in rural areas had higher risk of FI because they did not have direct access to food [24]. In this way, the analysis in this paper considered the following covariates: per capita family income (≤1, >1 and ≤2, or >2 minimum wages), based on the values in force during the sample reference period (BRL 954.00); household location (urban or rural); and the main places where food is purchased, including supermarkets, small markets, and fruit and vegetable markets.

### 2.7. Statistical Analysis

The sociodemographic profile of households was assessed for the country and macroregions. Additionally, the percentage distribution of food acquisition locations was calculated according to food security and levels of FI for the country and regions, and the significance between estimates was compared via a chi-square test (*p* value < 0.05). To evaluate the quality of purchased food according to acquisition location, the relationships between food groups and acquisition locations were compared between food-secure and food-insecure households.

Analyses were performed via the statistical program STATA 16.0 (https://www.stata.com) in survey mode, which accounts for the expanded sample, and confidence intervals (CIs) at the 5% significance level (95% CIs) were estimated to test statistical associations.

## 3. Results

Brazilian households across the different macroregions of the country present different sociodemographic profiles, particularly regarding income inequalities. The North and Northeast macroregions stand out for having the highest proportions of families with lower incomes, while the South and Southeast macroregions have the highest percentages of individuals with incomes exceeding twice the minimum wage (44.5% and 47.6% of the national population, respectively).

With respect to food acquisition, the majority of the Brazilian population makes food purchases at supermarkets, followed by small markets. However, this trend showed regional variations. In the North and Northeast regions, small markets predominate as the main acquisition location, while in the South, Southeast, and Central–West regions, supermarkets are more common. On the other hand, markets and fruit and vegetable stores have lower popularity, with their use varying between 8.0% and 19.6% across the different macroregions of the country (Table 2).

These study results indicate a difference in the percentage distribution of food acquisition locations according to levels of food security and FI across regions in Brazil. Nearly half of the food-secure families located in the North and Northeast regions obtained their food primarily from small markets. In contrast, families in the South, Southeast, and Central–West regions predominantly acquired food from supermarkets. When mild FI was present, food acquisition was greater in small markets among families in the North and Northeast. However, families with the same mild FI conditions residing in the South, Southeast, and Central–West regions predominantly acquired food from supermarkets. Compared to families with other levels of FI, families experiencing more severe FI levels (moderate/severe) presented a significantly higher preference for small markets as food acquisition locations, except for families in the Central–West region (Table 3).

Although the acquisition of food at markets, fruit and vegetable stores, and public markets did not differ significantly between food-secure families and those with different levels of FI across various macroregions, a significant proportion of families in the Northeast opted for these locations for food purchases, as presented in Table 3.

Table 4 presents a comparison of the patterns of acquisition for different food groups according to the places of acquisition in Brazil. The results indicate that two-thirds of the food groups assessed were acquired through purchases at supermarkets (rice, beans, vegetables and greens, fruits, poultry, eggs, milk and dairy products, biscuits, and sugary beverages). Small markets were particularly relevant for the acquisition of bread and red meat. Markets, fruit and vegetable stores, and public markets also played a significant role in the acquisition of vegetables and greens and fruits, accounting for 36.1% of fruit acquisitions and 34.0% of vegetable and greens acquisitions.

Table 5 shows that food-secure families went to supermarkets to purchase almost all analyzed food groups, except bread, which was more commonly purchased at small markets. For families with mild FI, although supermarkets were the preferred location for the acquisition of basic foods such as rice and beans, small markets were prominent for the acquisition of red meat, poultry, milk and dairy products, and bread. Markets, fruit and vegetable stores, and public markets also emerged as important places for purchasing fruits for families with mild FI. Among families with moderate/severe FI, 52.1% of rice purchases and 45.0% of bean purchases were made at small markets, while fruits, vegetables and greens, red meat, and fish were commonly acquired at street markets, fruit and vegetable stores, and public markets (*p* value < 0.05).

## 4. Discussion

The results of this study highlight the heterogeneity regarding different food acquisition locations in Brazil. Food-secure families, who enjoy regular and permanent access to adequate food in terms of quality and quantity, prefer supermarkets as their primary place for food acquisition. For families experiencing moderate/severe FI, whose usual food patterns are disrupted, and who experience reduced quality and quantity of food for all family members, small markets are more frequently used.

Previous studies conducted in Brazil to identify food acquisition locations and their impacts on nutrition [16,17] also identified supermarkets as the primary locations for food acquisition by families. However, this is the first study in Brazil to investigate the relationship between food acquisition locations and the level of FI among Brazilian families.

The dominant role of the supermarket sector in food retail is well established, and various national and regional chains operate throughout the country [25]. However, there are differences in the concentration and availability of supermarkets across regions [26], and socioeconomic and demographic characteristics can influence the presence and operation of these establishments. Regions with a higher per capita income are typically targeted for investment by major supermarket chains, while regions with a lower income may experience more limited availability [9,27].

Importantly, purchasing food at supermarkets does not guarantee quality in the population’s diet. Studies have shown a direct relationship between the frequency of supermarket purchases and the consumption of ultraprocessed foods [28]. Costa et al. (2013) demonstrated that the location where consumers acquire their food has a significant effect on diet quality: supermarket purchases are associated with higher consumption of processed and less healthy foods, while purchases at street markets are related to a diet rich in fresh foods [16]. Notably, ultraprocessed foods are a public health concern, as excessive consumption of these products is linked to various health issues, such as overweight, obesity, cardiovascular diseases, and type 2 diabetes [29,30].

This study also revealed that supermarkets were prominent in the acquisition of ultraprocessed foods, such as biscuits and sugary beverages, regardless of the food security/FI levels of the families. In their study, Machado et al. [17] analyzed the association between the type of food retail establishment and the consumption of ultraprocessed products in Brazil and noted that families with higher consumption of ultraprocessed products made a greater share of their total food purchases at supermarkets. The authors also highlighted that traditional retail establishments were associated with lower acquisition of ultraprocessed foods

With respect to the organization of the supermarket sector, there has been a strategic shift toward opening smaller stores distributed across various neighborhoods, which increases the competition with smaller food retail establishments. As a result, there has been a gradual reduction in traditional retail trade and, consequently, a decrease in commercial diversity in cities. Another related impact is the change in relationships with suppliers, farmers, and intermediaries, who now negotiate not with market vendors but with large retail corporations [26].

With respect to traditional retail trade, which includes street markets, small grocery stores, bakeries, butchers, etc., it is important to highlight that these establishments differ in the products they sell, as well as in their size and physical structure [31,32]. Additionally, the diversity among consumers who purchase food from these retail establishments includes differences in income, education level, and preferences, contributing to the sustainability of these small businesses [33]. These smaller establishments are often attractive due to their proximity to households, which allows them to remain economically viable through the sale of products in a more convenient manner (pricing), especially for segments of the population with lower purchasing power [34].

As evidenced by our results, families experiencing FI, particularly those with moderate/severe insecurity, showed a greater tendency to acquire food from small markets, especially in regions with lower economic levels (North and Northeast). A study conducted by GfK Custom Research Brazil in collaboration with the Brazilian Supermarket Association (ABRAS) in 2017 revealed that in Brazil, approximately 90% of small markets are family-owned, in contrast with the large supermarket chains characterized by standardization and monopolization. In terms of the most commonly purchased product categories, the dry grocery section accounted for 20.3% of purchases, followed by the butcher section, which accounted for 17.4% of sales [35].

When investigating the food purchase locations of families, regardless of their level of FI, small markets were used to purchase nearly all the food groups studied, except for vegetables and fruits. This situation contradicts the recommendations of the Dietary Guidelines for the Brazilian Population, which highlight natural foods as fundamental for a healthy and adequate diet [36]. The sale of vegetables and fruits in small markets is essential for ensuring access to a healthy and varied diet, especially for more vulnerable populations, who often rely on small markets to meet their daily food needs.

The results of this study also highlight the importance of markets as essential points for the acquisition of natural foods by Brazilian families. In the Northeast region, markets were prominent not only in the purchase of fruits and vegetables but also in their ability to serve both food-secure households and those at various levels of FI. This result emphasizes the vital role of markets in ensuring access to healthy and affordable foods, adapting to the specific needs of each area, and contributing to the diversity and quality of the local food supply. Strengthening markets, small grocery stores, and local producers could be an effective strategy to increase the availability of foods that promote healthier diets [37,38,39].

Regarding the limitations of this study, the household collective acquisition notebook, in which purchase locations and acquired foods were recorded, was filled out by the participants themselves, potentially introducing memory errors and inaccuracies. Importantly, POF data collection was conducted over 8 days by trained interviewers, which tends to minimize such inaccuracies. Nevertheless, owing to this limitation, it is crucial to conduct further studies to provide additional insights into this topic.

The strengths of this study include the insights it provides for understanding how food acquisition locations facilitate access to food in different contexts and contribute to an unequal food environment from the perspective of food access. This is the first study aimed at identifying food acquisition locations in relation to FI, where the location of acquisition is a crucial element of “access”. Another strength is that this is a national study based on the most recent food acquisition survey, which provides a sample that allows for regional comparisons and scenario differentiation, and it thus enables the formulation of targeted public policies.

## 5. Conclusions

This study revealed that, despite the predominance of supermarkets in Brazil, families experiencing FI frequently turn to small markets and street fairs for their food purchases. Additionally, it underscores the importance of these small-scale establishments in the North and Northeast regions of Brazil, which have higher concentrations of families with lower per capita incomes than other regions.

A notable finding is the preference for small-scale establishments for purchasing fruits and vegetables, particularly among families with moderate to severe FI. This highlights the potential to integrate and support small market vendors and street fairs as a strategy to address FI and promote adequate and healthy nutrition for families struggling to secure their right to adequate food.

In this context, analyzing food acquisition locations becomes essential, as it can guide the formulation of public policies that integrate small vendors and local producers, fostering local economic development and improving family nutrition.

Currently, there are no widely consolidated actions in Brazil in this regard. Recently, on 16 October 2024, the Federal Government published the National Food Supply Plan (in Portuguese, Alimento no Prato) [40]. This is an important initiative to strengthen policies that combat hunger and promote food sovereignty in Brazil. The Alimento no Prato Plan, comprising 29 initiatives, aims to enhance existing programs and implement new actions to ensure access to healthy food from production to consumption. According to this new Federal Government initiative, small markets, grocery stores, butcher shops, and fish markets will also be requalified to provide healthy food in areas of low availability through the creation of the Healthy Retail Network, which will supply these points of sale with products from family farmers or wholesale food distribution centers (Ceasas).

This model will not only strengthen small vendors by providing them with healthier food options, but will also encourage the inclusion of small producers in the supply chain, thereby stimulating the local economy. Additionally, supporting and expanding the guidelines of the Dietary Guidelines for the Brazilian Population will help promote healthier eating practices and reduce the negative impacts of poor diets, such as obesity and chronic diseases. Thus, the integration of public policies, small vendors, and local producers represents a promising solution to address the challenges of food insecurity in Brazil.

## Figures and Tables

**Table 1:** Definitions of analyzed food groups in Brazil in 2017–2018.

Food Groups	Included Foods
Rice	White rice, polished rice, parboiled rice
Beans	Black-eyed peas, black beans, cowpeas, other beans
Vegetables and greens	Cabbage, chard, kale, parsley, watercress, lettuce, cauliflower, tomato, bell pepper, beetroot, eggplant, carrot, okra, pumpkin, jiló (a type of bitter eggplant), string beans, onion, chayote, other vegetables and greens
Fruits	Banana, orange, apple, lemon, melon, watermelon, pineapple, avocado, mango, guava, strawberry, grape, pear, papaya, persimmon, tangerine, other fruits
Red meats	First-cut beef, second-cut beef
Fish	Fresh fish, frozen filleted fish, freshwater fish, saltwater fish
Poultry	Chicken wing, chicken breast, chicken thigh, chicken back, other chicken cuts
Eggs	Chicken egg
Milk and dairy products	Pasteurized cow’s milk, fresh cow’s milk, cream, condensed milk, whole milk powder, skim milk powder, other dairy products
Bread	Industrialized sliced bread, cheese bread, corn bread, whole grain bread, sweet bread, other breads
Biscuits	Filled/sweet cookies, savory cookies
Sugary drinks	Soft drinks, powdered fruit juice, bottled fruit juice, energy drinks, other beverages

**Table 2:** Descriptive analysis of households with prevalences (%) and corresponding confidence intervals (CI95%) for sociodemographic and economic variables In Brazil in 2017–2018.

Sociodemographic Characteristics	Brazil		North		Northeast	
	%	95% CI	%	95% CI	%	95% CI
Monthly per capita income (minimum wage)						
≤1	29.9	(29.0; 30.8)	58.5	(55.6; 61.4)	49.7	(48.2; 51.2)
>1 e ≤ 2	34.0	(33.1; 34.9)	26.5	(24.1; 28.5)	31.5	(30.3; 33.0)
>2	36.1	(34.9; 37.4)	15.2	(13.2; 17.5)	18.7	(17.2; 20.2)
Area						
Urban	88.4	(87.9; 89.0)	81.3	(79.5; 83.0)	79.2	(77.7; 80.6)
Rural	11.6	(11.0; 12.1)	18.7	(17.0; 20.5)	20.8	(19.4; 22.3)
Place of purchase						
Supermarkets	45.0	(44.1; 46.0)	33.8	(31.1; 36.5)	28.2	(27.0; 29.5)
Small markets	36.1	(35.3; 37.0)	48.0	(46.5; 50.4)	47.0	(45.8; 48.2)
Street fair/fruit and vegetable stores/public markets	14.5	(14.0; 15.0)	12.3	(10.6; 14.2)	19.6	(18.8; 20.5)
Others	4.3	(4.1; 4.5)	5.9	(5.3; 6.6)	5.1	(4.7; 5.5)
	**South**		**Southeast**		**Center–West**	
	**(%)**	**95% CI**	**(%)**	**95% CI**	**(%)**	**95% CI**
Monthly per capita income (minimum wage)						
≤1	19.8	(18.4; 21.2)	17.1	(15.7; 18.7)	21.6	(19.6; 23.8)
>1 e ≤ 2	35.7	(34.0; 37.4)	35.2	(33.1; 37.4)	37.1	(34.2; 40.0)
>2	44.5	(42.3; 46.9)	47.6	(45.3; 50.0)	41.3	(37.6; 45.1)
Área						
Urban	94.8	(94.0; 95.5)	87.5	(86.3; 88.7)	92.0	(91.2; 92.8)
Rural	5.2	(4.5; 6.0)	12.5	(11.3; 13.7)	8.0	(7.2; 8.9)
Place of purchase						
Supermarkets	53.1	(51.4; 54.8)	52.8	(50.6; 55.1)	49.7	(46.7; 52.7)
Small markets	29.8	(28.4; 31.3)	33.4	(31.4; 35.6)	29.7	(27.4; 32.1)
Street fair/fruit and vegetable stores/public markets	14.1	(13.1; 15.2)	8.0	(7.3; 8.7)	15.4	(14.1; 16.8)
Others	2.9	(2.7; 3.2)	5.7	(5.1; 6.4)	5.2	(4.7; 5.7)

Minimum wage (BRL 954.00). 95% CI: 95% confidence interval. *p* < 0.05 (Pearson chi-square test).

**Table 3:** Percentage distribution (%) and corresponding 95% confidence intervals (CI) of food acquisition locations by level of food security and food insecurity among families by macroregion of Brazil in 2017–2018.

Variables	Supermarkets		Small Markets		Street Fairs/Fruit and Vegetable Stores/Public Markets		Others	
	%	95% CI	%	95% CI	%	95% CI	%	95% CI
North *								
SA	40.2	(36.4; 44.1)	41.8	(38.7; 44.9)	13.1	(10.3; 16.5)	4.9	(4.1; 5.8)
Mild FI	33.1	(29.0; 37.6)	48.3	(44.7; 52.0)	12.7	(9.7; 16.4)	5.8	(4.9; 7.0)
Moderate/ Severe FI	25.1	(21.8; 28.7)	56.8	(53.3; 60.2)	10.7	(9.2; 12.3)	7.4	(6.3; 8.7)
Northeast *								
SA	32.0	(30.2; 34.0)	43.2	(41.6; 44.8)	20.0	(18.9; 21.2)	4.7	(4.3; 5.2)
Mild FI	25.9	(24.2; 27.6)	49.5	(47.8; 51.2)	19.3	(18.1; 20.6)	5.3	(4.8; 5.8)
Moderate/ Severe FI	21.2	(19.2; 29.5)	54.0	(51.9; 56.0)	19.1	(17.6; 20.6)	5.7	(4.9; 6.7)
South *								
SA	54.9	(53.0; 56.7)	29.7	(26.3; 29.6)	14.4	(13.2; 15.8)	2.8	(2.5; 3.1)
Mild FI	51.9	(47.8; 54.2)	32.4	(29.9; 34.9)	13.4	(11.4; 15.6)	3.2	(2.6; 3.9)
Moderate/ Severe FI	43.2	(36.4; 45.3)	42.2	(37.9; 46.6)	13.1	(10.8; 15.8)	3.9	(3.1; 5.0)
Southeast *								
SA	53.6	(51.2; 55.9)	32.4	(30.3; 40.5)	8.2	(7.4; 9.0)	5.8	(5.1; 6.7)
Mild FI	51.4	(46.9; 55.8)	36.3	(29.9; 34.9)	7.0	(5.6; 8.5)	5.4	(4.2; 6.8)
Moderate/ Severe FI	43.2	(35.2; 51.5)	44.1	(36.3; 52.2)	8.0	(5.0; 10.7)	4.7	(3.2; 6.7)
Center–West *								
SA	52.0	(48.5; 55.5)	27.2	(24.7; 29.8)	15.6	(13.9; 17.5)	5.2	(4.6; 5.8)
Mild FI	47.5	(43.8; 51.2)	33.2	(30.3; 36.6)	13.8	(11.6; 16.3)	5.5	(4.5; 6.7)
Moderate/ Severe FI	38.2	(32.3; 44.3)	39.6	(34.4; 45.0)	17.7	(13.9; 22.3)	4.5	(3.4; 6.0)

95% CI: 95% confidence interval. * *p* < 0.05 (Pearson chi-square test).

**Table 4:** Percentage distribution (%) and corresponding confidence intervals (95% CI) of food group acquisition by acquisition location in Brazil in 2017–2018.

Food Groups	Supermarkets		Small Markets		Street Fairs/Fruit and Vegetable Stores/ Public Markets		Others	
	%	95% CI	%	95% CI	%	95% CI	%	95% CI
Rice	50.6	(49.0; 52.1)	38.9	(37.3; 40.5)	9.8	(8.9; 10.8)	0.7	(0.5; 1.0)
Beans	50.6	(48.9; 52.3)	32.9	(31.3; 34.5)	13.1	(12.1; 14.3)	3.4	(2.7; 4.1)
Vegetables and greens	40.0	(38.5; 41.1)	18.0	(17.1; 18.9)	34.0	(32.8; 35.2)	8.2	(7.6; 8.9)
Fruits	42.4	(41.0; 43.9)	16.2	(15.3; 17.2)	36.1	(34.8; 37.5)	5.2	(4.8; 5.7)
Red meats	44.5	(43.2; 45.8)	46.9	(45.7; 48.2)	7.2	(6.6; 7.9)	1.4	(1.1; 1.7)
Fish	24.7	(22.5; 27.1)	31.0	(28.7; 33.3)	32.6	(30.1; 35.3)	11.6	(10.0; 13.5)
Poultry	45.6	(44.4; 47.1)	40.6	(39.3; 42.0)	10.0	(9.3; 10.7)	3.6	(3.3; 4.0)
Eggs	31.4	(29.9; 32.9)	31.3	(29.9; 32.7)	13.6	(12.6; 14.7)	23.7	(21.8; 25.6)
Milk and dairy products	40.8	(35.6; 38.9)	37.3	(35.8; 38.9)	8.8	(8.1; 9.6)	13.1	(11.9; 14.3)
Bread	18.0	(17.2; 18.8)	76.5	(75.5; 77.4)	2.9	(2.6; 3.3)	2.6	(2.3; 3.0)
Biscuits	52.3	(50.6; 53.9)	37.2	(35.7; 38.8)	9.3	(8.2; 10.4)	1.2	(0.9; 1.6)
Sugary drinks	51.4	(49.7; 53.0)	37.7	(36.2; 39.2)	7.5	(6.6; 8.4)	3.5	(3.0; 4.0)

95% CI: 95% confidence interval. *p* < 0.05 (Pearson chi-square test).

**Table 5:** Percentage distribution (%) and corresponding 95% confidence intervals (CI95%) of food group purchases by acquisition location and levels of food security and food insecurity in Brazil in 2017–2018.

Food Groups	Supermarkets		Small Markets		Street Fairs/Fruit and Vegetable Stores/ Public Markets		Others	
	%	95% CI	%	95% CI	%	95% CI	%	95% CI
SA *								
Rice	57.2	(55.3; 59.1)	33.6	(31.7; 35.5)	8.6	(7.5; 9.9)	0.5	(0.3; 0.9)
Beans	56.1	(53.9; 58.2)	28.6	(26.6; 30.6)	12.2	(10.7; 13.9)	3.1	(2.5; 4.0)
Vegetables and greens	42.7	(41.2; 44.3)	16.4	(15.4; 17.5)	32.9	(31.5; 34.3)	8.0	(7.2; 8.8)
Fruits	45.8	(44.1; 47.5)	15.3	(14.2; 16.5)	34.4	(32.8; 36.1)	4.5	(4.1; 5.0)
Red meats	47.9	(46.2; 48.5)	44.1	(42.5; 45.7)	6.6	(5.7; 7.5)	1.4	(1.1; 1.8)
Fish	33.4	(30.1; 37.0)	31.7	(28.7; 34.8)	26.8	(23.8; 29.9)	8.1	(6.7; 9.7)
Poultry	51.2	(49.4; 52.9)	36.5	(34.8; 38.2)	8.9	(8.0; 9.8)	3.5	(3.0; 3.9)
Eggs	35.7	(33.9; 37.6)	25.3	(23.8; 27.0)	14.9	(13.5; 16.4)	24.1	(21.9; 26.4)
Milk and dairy products	44.3	(42.5; 46.2)	35.0	(33.2; 36.9)	8.1	(7.3; 8.9)	12.6	(11.3; 14.0)
Bread	22.2	(21.2; 23.3)	72.9	(71.8; 74.1)	2.8	(2.4; 3.3)	2.0	(1.7; 2.4)
Biscuits	56.3	(54.3; 58.3)	33.7	(31.9; 35.9)	8.7	(7.5; 10.4)	1.1	(0.7; 1.8)
Sugary drinks	54.9	(52.9; 56.8)	34.0	(32.2; 35.8)	7.6	(6.5; 8.9)	3.5	(2.9; 4.3)
Mild FI *								
Rice	48.9	(46.1; 51.7)	40.3	(37.7; 42.9)	10.1	(8.7; 11.8)	0.7	(0.4; 1.1)
Beans	48.4	(45.7; 51.2)	34.5	(32.0; 37.1)	13.5	(11.7; 15.6)	3,5	(2.4; 5.0)
Vegetables and greens	36.0	(33.7; 38.4)	20.2	(18.6; 21.9)	35.5	(33.3; 37.8)	8.3	(7.2; 9.5)
Fruits	35.7	(33.2; 38.2)	17.9	(16.2; 19.8)	40.0	(37.7; 42.5)	6.3	(5.5; 7.2)
Red meats	42.2	(39.8; 44.7)	49.3	(46.9; 51.7)	7.4	(6.6; 8.3)	1.1	(0.7; 1.6)
Fish	20.5	(17.4; 23.9)	31.2	(27.6; 35.0)	35.7	(31.5; 40.2)	12.5	(10.1; 15.5)
Poultry	41.7	(39.5; 44.0)	43.5	(41.3; 45.7)	11.2	(10.0; 12.6)	3.5	(3.0; 4.2)
Eggs	27.7	(25.3; 30.3)	37.4	(34.8; 40.2)	11.1	(9.5; 12.9)	23.8	(20.8; 27.0)
Milk and dairy products	35.8	(33.5; 38.2)	39.5	(37.0; 42.0)	10.5	(8.6; 11.8)	14.6	(12.8; 16.7)
Bread	13.1	(11.9; 14.3)	80.8	(79.4; 82.3)	2.9	(2.4; 3.5)	3.2	(2.5; 3.9)
Biscuits	48.3	(45.5; 51.0)	40.3	(37.8; 42.8)	10.1	(8.3; 12.3)	1.3	(0.9; 1.8)
Sugary drinks	47.3	(44.3; 50.2)	42.4	(39.7; 45.2)	7.0	(5.5; 9.1)	3.3	(2.7; 4.0)
Moderate/Severe FI *								
Rice	34.0	(31.2; 36.8)	52.1	(48.8; 55.4)	12.7	(10.7; 15.2)	1.2	(0.6; 2.3)
Beans	35.6	(32.3; 38.6)	45.0	(41.8; 48.3)	15.7	(13.5; 18.1)	3.9	(2.8; 5.5)
Vegetables and greens	26.4	(23.7; 29.4)	24.6	(22.3; 27.2)	38.8	(35.7; 42.0)	10.1	(8.4; 12.1)
Fruits	25.3	(22.2; 28.6)	21.4	(18.9; 24.2)	43.3	(39.9; 46.9)	10.0	(8.3; 11.9)
Red meats	29.6	(26.9; 32.4)	58.2	(55.3; 61.1)	10.3	(9.1; 11.8)	1.9	(1.3; 2.7)
Fish	9.9	(7.8; 12.6)	28.9	(25.0; 33.1)	42.3	(37.3; 47.5)	18.8	(19.9; 23.4)
Poultry	30.8	(28.0; 33.7)	52.5	(49.6; 55.4)	12.1	(10.5; 14.0)	4.6	(3.7; 5.7)
Eggs	18.0	(15.8; 20.5)	47.7	(44.2; 51.1)	12.6	(10.5; 14.9)	21.7	(18.2; 25.6)
Milk and dairy products	28.0	(24.7; 31.5)	47.9	(43.6; 52.3)	11.3	(9.2; 13.9)	12.8	(9.9; 16.3)
Bread	7.7	(6.7; 8.9)	84.5	(82.7; 86.1)	3.5	(2.7; 4.5)	4.3	(3.3; 5.5)
Biscuits	38.0	(34.6; 41.5)	51.2	(47.8; 54.7)	9.7	(7.9; 11.8)	1.1	(0.7; 1.6)
Sugary drinks	36.2	(32.5; 40.1)	53.1	(49.0; 57.1)	7.2	(5.7; 9.1)	3.5	(2.5; 4.8)

95% CI: 95% confidence interval. * *p* < 0.05 (Pearson chi-square test).

## Data Availability

This study analyzed publicly available datasets. These data can be accessed at https://www.ibge.gov.br/estatisticas/sociais/populacao/24786-pesquisa-de-orcamentos-familiares-2.html?=&t=microdados (accessed on 5 January 2022).
